# Regular Cold‐Water Immersion Following HIIT Does Not Affect Intramuscular Adaptation Markers, Inflammatory Profile or Endurance Performance

**DOI:** 10.1111/sms.70241

**Published:** 2026-02-25

**Authors:** Elvis S. Malta, José Cesar Rosa Neto, Wladimir R. Beck, Anabelle S. Cornachione, Rodrigo A. B. de Poli, Emilly Sigoli, Alessandro M. Zagatto

**Affiliations:** ^1^ Laboratory of Physiology and Sport Performance—LAFIDE, Department of Physical Education, School of Sciences São Paulo State University—UNESP Bauru Brazil; ^2^ Immunometabolism Research Group, Department of Cell and Developmental Biology University of São Paulo—USP São Paulo Brazil; ^3^ Laboratory of Endocrine Physiology and Physical Exercise, Department of Physiological Sciences Federal University of São Carlos—UFSCAR São Carlos Brazil; ^4^ Muscle Physiology and Biophysics Laboratory, Department of Physiological Sciences Federal University of São Carlos São Carlos Brazil; ^5^ College of Health and Life Sciences Hamad Bin Khalifa University Doha Qatar

**Keywords:** cryotherapy, ice bath, ice therapy, interval training, muscle adaptation

## Abstract

The study aimed to investigate the effects of 5 weeks of post‐exercise cold‐water immersion (CWI) following high‐intensity interval training (HIIT) sessions on the satellite cell pool, muscle content of inflammatory markers, muscle expression of peroxisome proliferator‐activated receptor‐γ coactivator 1‐α (PGC‐1α), maximal oxygen uptake (V̇O_2_max), and running performance. Sixteen healthy males completed baseline assessments, including muscle biopsies, a graded exercise test for V̇O_2_max determination, and a constant work‐rate (CWR) running test to assess time to task failure (TTF). Participants were ranked according to V̇O_2_max and randomly allocated to either a training‐only control group (*n* = 7) or a CWI group (*n* = 9), which underwent CWI (11.2°C ± 0.2°C for 15 min) following each HIIT session. The HIIT program consisted of three weekly sessions 5–8 × 2‐min bouts at 95% V̇O_2_max. At the end of weeks four and five, all participants repeated the same sequence of assessments. Training increased V̇O_2_max values, TTF at CWR, satellite cell pool, PGC‐1α content, and induced changes in muscle morphology (connective tissue), as indicated by a main effect of time (*p* ≤ 0.031); none of the analyzed variables showed a main effect of condition (*p* ≥ 0.098) or interaction (*p* ≥ 0.088). No significant alterations were observed in inflammatory markers over time (*p* ≥ 0.395) and condition (*p* ≥ 0.115). In conclusion, 5 weeks of post‐exercise CWI following HIIT did not influence the satellite cell pool, muscle inflammation status, muscle PGC‐1α content, muscle morphological, V̇O_2_max, or running performance.

## 1 Introduction

Cold‐water immersion (CWI) is widely used by athletes and non‐athletes and involves whole‐ or partial‐body immersion in cold water. Typical protocols range from approximately 10°C to 15°C for durations of 5 to 20 min [1]. CWI is commonly applied to enhance post‐exercise recovery [2] and has also been used as a muscle pre‐cooling strategy before sporting events [3]. The primary physiological effects of CWI are largely attributed to vasoconstriction induced by muscle cooling, with hydrostatic pressure playing a secondary role [2]. Vasoconstriction may acutely reduce metabolic activity [4], hormone secretion [5], immune cells infiltration [6], and limb blood flow [7, 8, 9]. Although consensus is lacking, these responses have been associated with reductions in markers of exercise‐induced muscle damage, systemic and intramuscular inflammation [5, 10, 11, 12, 13, 14], delayed onset muscle soreness [10, 15, 16], and with maintenance of muscle function [14, 17, 18], collectively suggesting an improved recovery status.

Given these acute effects, CWI has been proposed as a beneficial adjunct to training, with potential to improve training quality and enhance muscle adaptations and performance [19]. However, recent evidence indicated that regular post‐exercise CWI may negatively affect training adaptations in a task‐dependent manner [20]. Specifically, when applied consistently during resistance training, post‐exercise CWI can blunt training‐induced protein synthesis, anabolic signaling, and satellite cell activation [21, 22, 23], thereby attenuating hypertrophy and strength gains [23]. In contrast, although fewer studies are available, regular CWI does not appear to impair aerobic performance improvements when used following endurance training [24, 25, 26, 27]. Nevertheless, its effects on cardiorespiratory fitness indices and intramuscular oxidative adaptations remain unclear.

Yamane et al. [27] reported attenuated endurance performance and maximal oxygen uptake (V̇O_2_max) following 4–6 weeks of post‐exercise CWI compared with a control condition using a crossover design. In contract, Broatch et al. [24] applied CWI following sprint interval training for 6 weeks and observed no differences in endurance performance (i.e., incremental test or time‐trial) or V̇O_2_max compared with a control condition in a parallel‐group design. Similarly, Halson et al. [25] combined CWI with sprint interval training and sport‐specific cycling efforts over 5.5 weeks and found no differences in endurance performance adaptations between groups.

Incorporating intramuscular markers may help clarify the mechanisms underlying these conflicting findings. Acute CWI has been shown to increase the expression of peroxisome proliferator‐activated receptor‐γ coactivator‐1α (PGC‐1α) mRNA [28], a key regulator of mitochondrial biogenesis and oxidative adaptations [29]. However, neither acute nor chronic exercise appears to alter PGC‐1α protein content [24, 30]. Accordingly, the influence of repeated CWI exposure on PGC‐1α protein expression and long‐term intramuscular adaptations remains to be determined.

Although satellite cell regulation has not been a primary focus of endurance training studies involving CWI, this pathway may contribute to long‐term adaptations. Evidence from resistance training studies indicates that acute post‐exercise CWI can reduce satellite cells activation, as reflected by Paired Box Protein 7 (PAX‐7) or Neural Cell Adhesion Molecule (NCAM) expression [23]. Satellite cells play a critical role in muscle repair, remodeling, and long‐term adaptation and are regulated by hormonal signaling and exercise‐induced inflammation [31, 32]. Thus, repeated attenuation of satellite cell pool activation may influence endurance‐related adaptations, including maintenance if the satellite cell pool and muscle resilience [33]. In addition, acute CWI following endurance exercise has been shown to modulate inflammatory responses [5, 13]. Therefore, if CWI consistently blunts post‐exercise inflammatory signaling (e.g., IL‐6, TNFα, and IL‐10) and associated satellite cell activation responses, these effects may interact with key molecular pathways, such as PGC‐1α‐mediated oxidative signaling, potentially constraining intramuscular, morphological and cardiorespiratory adaptations over time.

Therefore, the aim of this study was to determine whether 5 weeks of post‐exercise CWI would attenuate intramuscular and cardiorespiratory adaptations, as reflected by changes in the satellite cell pool, inflammatory markers (interleukin [IL]‐6, IL‐10, and tumor necrosis factor alpha [TNFα]), PGC‐1α levels, V̇O_2_max, and running performance.

## 2 Methods

### 2.1 Participants and Ethics Approval

Sixteen healthy male volunteers (aged 25 ± 6 years) participated in this study. All participants reported no history of vascular diseases, metabolic disorders, or musculoskeletal injuries within the previous 6 months. Female participants were not recruited due to the additional complexity in study design required to account for sex‐specific factors (e.g., menstrual cycle, hormonal contraceptive use, menopausal status), recruitment challenges associated with studies involving muscle biopsy, and concerns about performance variability [34]. Prior to participation, all volunteers were informed about the study's procedures, potential risks, and benefits, and provided written informed consent. The study was approved by the Human Research Ethics Committee of the School of Sciences, São Paulo State University—UNESP (CAAE: 82797718.8.0000.5398/approval protocol: 2538336) and conducted in accordance with the Declaration of Helsinki [35].

### 2.2 Experimental Design

This investigation was a randomized, controlled trial with independent groups. Initially, skeletal muscle samples were collected by biopsy after approximately 5 days of rest (i.e., restriction on physical activity). On the second visit (approximately 3 days later), participants underwent a familiarization session during which all evaluation procedures were simulated (e.g., submaximal incremental and continuous running efforts). On the third and fourth visits were carried out a graded exercise test (GXT) to determine V̇O_2_max and constant work rate (CWR) running effort until exhaustion to assess time to task failure (TTF). In the present study, these initial evaluations will be referred to as “baseline”.

Sixteen volunteers were randomly assigned into two groups using a stratified randomization method using the GXT data results [36]. One group underwent CWI following each training session (*n* = 9), while the control group completed the training program without CWI (*n* = 7). After group allocation, the cardiorespiratory and anthropometric characteristics of the CWI and control groups were comparable. V̇O_2_max values were 42.0 ± 3.9 and 42.4 ± 5.7 mL kg^−1^ min^−1^ (*p* = 0.886), body mass was 80.7 ± 12.6 and 83.2 ± 20.7 kg (*p* = 0.768), and height was 178.2 ± 4.8 and 178.0 ± 3.8 cm (*p* = 0.941), respectively.

The HIIT program lasted 5 weeks plus one additional session day, totaling 16 sessions, three sessions per week, with one final session in the sixth week. The five‐week HIIT period was selected to allow sufficient time to detect potential increases in the satellite cell pool [32] and to elicit meaningful cardiorespiratory and running‐performance adaptations [37]. It was structured into two phases: a 4‐week overload phase (12 sessions), during which training volume and, when necessary, intensity (see more details in HIIT sessions) were increased, followed by a tapering phase lasting 1 week and 1 day (4 sessions), characterized by reduced training volume. After each training session, participants either underwent CWI (intervention group) or passive recovery (control group), according to group allocation. All tests and training sessions were performed on a motorized treadmill (ATL, Inbramed, Inbrasport, Porto Alegre, Brazil) set at a constant 1% incline.

**FIGURE 1:**
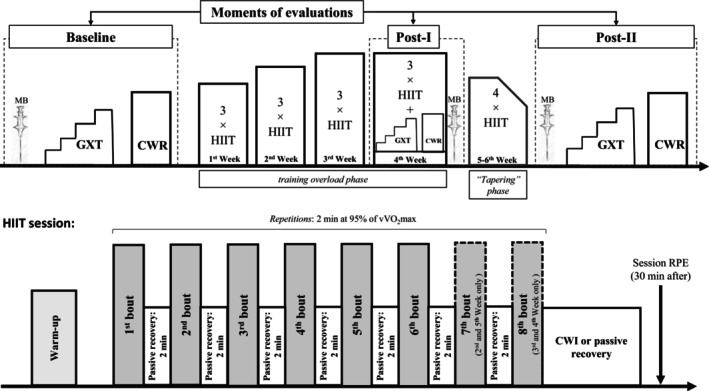
Experimental design of the study. Experimental design of the study. The first timeline presents the evaluation moments (baseline, Post‐I, and Post‐II), including the GXT and CWR tests, the three biopsy time points, and the progression of the HIIT protocol over time (training overload phase and tapering phase). The second timeline presents a single HIIT session, detailing the number of repetitions within the training program, training intensity, work‐to‐rest duration, the timing of RPE assessment (used for session‐load calculation), and the point at which the intervention was applied. CWI, cold‐water immersion; CWR, constant work rate; GXT, graded exercise test; HIIT, high‐intensity interval training; MB, muscle biopsy; RPE, rate of perceived exertion.

The exercise tests (GXT and CWR) and skeletal muscle sampling procedures were repeated at the end of the fourth week (Post‐I) and again at the end of the training program (Post‐II). The experimental design is illustrated in Figure 1.

#### 2.2.1 Exercise Protocols

##### 2.2.1.1 Graded Exercise Test

Prior to testing, participants completed a standardized 5‐min warm‐up at a speed of 8 km h^−1^. During the GXT, they wore a safety harness attached to the ceiling to ensure maximal effort while minimizing the risk of falls or injury. The GXT started at 8 km h^−1^ with the treadmill gradient fixed at 1% [38]. The running velocity was increased by 1.5 km h^−1^ every 2‐min stage until volitional exhaustion, even with strong verbal encouragement [39]. Five minutes after completing the GXT, the participants performed a verification trial at a supramaximal speed (5% higher than the velocity achieved at V̇O_2_max) until volitional cessation [40]. This protocol allowed to determine the V̇O_2_max and the minimum velocity at which V̇O_2_max was achieved (vV̇O_2_max) (see Data analysis section for further details).

##### 2.2.1.2 Constante Work Rate (CWR) Running Effort

The CWR was performed at vV̇O_2_max until volitional exhaustion, with cardiorespiratory responses recorded throughout the effort. The TTF and cardiorespiratory parameters at exhaustion were determined using data from the final 30 s of exercise. For the CWR, evaluations at all time points (baseline, Post‐I, and Post‐II) were conducted at the same velocity corresponding to the baseline vV̇O_2_max.

##### 2.2.1.3 High‐Intensity Interval Training Sessions

Prior to each exercise session, participants completed a standardized warm‐up like that performed before the GXT. Each HIIT session consisted of 5–8 2‐min bouts at 95% of vV̇O_2_max. During the training overload phase, the number of bouts progressed with 6 in week 1, 7 in week 2, and 8 in weeks 3 and 4. During the tapering phase, the number of bouts decreased to 7, 6, and 5 in week 5, and 5 bouts in the final session. These effort bouts were interspaced by 2 min of passive recovery [41, 42]. The HR responses were continuously monitored using a V800 HR monitor (Polar, Kempele, Finland). Training intensity was adjusted by 5% when necessary to ensure that the exercise duration remained within the target “red zone” HR range (approximately 95% of the HR achieved during the GXT) [41, 42]. All training sessions were continuously monitored by the research team using HR monitors. If, during a given HIIT session, participants failed to reach the red zone in more than 50% of the bouts, the exercise intensity for the subsequent session was increased by 5% (i.e., the next session's bouts were performed at 100% of the initial vV̇O_2_max until the following GXT evaluation).

Thirty minutes after each session, participants rated their perceived exertion using the RPE CR‐10 scale. The session's internal load was quantified by multiplying the RPE score by the duration of the exercise session in minutes [43].

#### 2.2.2 Cold‐Water Immersion and Passive Recovery Sessions

The CWI was performed immediately after each training session (within approximately 40 s). Participants were seated in an immersion bath containing approximately 150 L of water at 11.2°C ± 0.2°C for 11 min [44]. During the CWI sessions, volunteers remained seated with hips flexed at 90°, legs fully extended and immersed up to the waist (water depth ≈25 cm). Water temperature was regulated using an auto‐cooling system controlled by a digital thermostat (TIC17RGTi, ES Full Gauge, USA), which activated whenever the temperature increased by 0.1°C. No ice was added to the water to avoid local temperature fluctuations. Conversely, the control group remained seated for 11 min at room temperature (20.7°C ± 1.5°C). Passive recovery was chosen because it provides a neutral control condition without adding physiological stimuli (inactive control) [45]. All CWI and passive recovery sessions were supervised and completed by the participants.

#### 2.2.3 Data Collection and Analyses

##### 2.2.3.1 Gas Exchange, Ventilatory and Physiological Variables

During GXT, verification trial, and CWR running tests, gas exchange and ventilatory data were measured breath‐by‐breath using a stationary metabolic cart (Quark PFT, Cosmed, Rome, Italy). The gas analyzer was calibrated using a sample of known gases (5% CO_2_ and 16% O_2_) and a 3‐L syringe (Hans Rudolf, Kansas City, MO), according to the manufacturer's recommendations. For analysis of gas exchange and ventilatory variables, outliers were first manually removed. Then, moving averages were calculated every five data points and interpolated to 1 s using the OriginPro 8.0 (Origin Lab Corporation, Massachusetts). Heart rate (HR) was measured beat‐by‐beat using a transmitter belt (Wireless HR Monitor, COSMED) coupled to the metabolic cart. For V̇O_2_max determination, the average oxygen uptake (V̇O_2_) during the final 30 s of each stage was calculated, and V̇O_2_max was defined as the highest average V̇O_2_ obtained, with a posteriori confirmation. The primary criterion for confirming V̇O_2_max was the presence of a plateau in V̇O_2_, defined as an increase of no more than 2.1 mL kg^−1^ min^−1^ between the final two stages of the GXT [46]. In the absence of a plateau, the highest V̇O_2_ achieved during the GXT was accepted as V̇O_2_max only if it differed by ≤ 1 mL kg^−1^ min^−1^ from the peak V̇O_2_ recorded during the verification trial [40]. If a discrepancy greater than 1 mL kg^−1^ min^−1^ was observed between tests, the GXT was repeated on a separate day; this was required for only one participant. The minimum velocity at which V̇O_2_max was achieved was recorded as vV̇O_2_max [47] and adjusted, when necessary (i.e., exercise stage that was not completed), according to the method proposed by Kuipers et al. [48]. In the aforementioned scenario, the adjusted value is derived by adding the velocity of the last fully completed stage to the product of the stage increment and the proportional duration completed within the prematurely terminated stage.

During CWR running effort, in addition to V̇O_2_, carbon dioxide production (V̇CO_2_), ventilation (V̇E), HR, and respiratory exchange ratio (RER) responses were also recorded using the same metabolic cart.

Moreover, capillary blood samples (25 μL) were collected from the earlobe 3 and 5 min after both the GXT and CWR tests. The samples were analyzed in an automated electrochemical analyzer (YSI 2900, Yellow Springs Instruments, Yellow Springs, USA) to determine the peak blood lactate concentrations, defined as the highest lactate value recorded following the tests. Additionally, the rate of perceived exertion (RPE) was assessed immediately after each test using the Borg 6–20 scale [49].

##### 2.2.3.2 Muscle Samples Collection

Vastus lateralis muscle biopsies were performed at three points: baseline, Post‐I (i.e., after 4 weeks) and Post‐II (i.e., after 5 weeks). The Post‐I and Post‐II biopsies were conducted 24 h after a training session, whereas the baseline biopsies were collected following a rest period. After local anesthesia with 2% lidocaine, a superficial incision (≈1.0 cm) was made, and a Bergström needle with suction was used to extract approximately 100 mg of muscle tissue. The muscle tissue sample was immediately sectioned into two equal fragments (≈50 mg each) for subsequent analyses. One fragment, representing the best‐preserved portion, was rolled in hydrated magnesium silicate and rapidly frozen in liquid nitrogen for subsequent immunofluorescence analysis. The second fragment was directly frozen in liquid nitrogen for later enzyme‐linked immunosorbent assay (ELISA). All samples were stored at −80°C in an ultra‐low temperature freezer until analysis. Following the biopsy, the incision site was dressed with a polyester‐filament micropore suture, covered with waterproof sterile dressing and bandage, and kept in place for approximately 1 h to prevent edema.

##### 2.2.3.3 Histology, Immunofluorescence Assay and ELISA

For histological evaluation, frozen muscle sections (6 μm) were stained with hematoxylin and eosin (H&E) to assess morphological characteristics, including the presence of central nuclei, connective tissue expansion, basophilic cells, fiber splitting, necrosis, and inflammatory infiltration. Images were captured using a Zeiss Vert. A1 light microscope equipped with AxioVision Rel 4.8 software (Zeiss, Jena, Thuringia, Germany) and a 40× objective lens. A semi‐quantitative analysis was subsequently performed.

Immunofluorescence analysis was employed to evaluate the quantification of PAX‐7 and PGC‐1α in skeletal muscle tissue. Samples were sectioned at a thickness of 6 μm using a cryostat (Leica CM 1850 UV, Wetzlar, Germany) and mounted onto histological slides. The section was incubated with primary monoclonal antibodies against PAX‐7 and PGC‐1α (Santa Cruz Biotechnology, USA), as well as a primary antibody for laminin (ABCAM, USA). Subsequently, appropriate fluorescent secondary antibodies were applied: anti‐PAX‐7 (Alexa Fluor Goat anti‐mouse IgG1 488), anti‐PGC‐1α (Alexa Fluor Goat anti‐mouse IgG2a 488), and anti‐laminin (Alexa Fluor Goat anti‐rabbit IgG 647). Nuclear staining was performed using 4′,6‐diamidino‐2‐phenylindole (DAPI). Slides were sealed and stored for further analysis.

Images were captured using a Leica SP5 confocal microscope (Leica Microsystems GmbH, Germany), selecting three random fields at 20× magnification from areas with preserved tissue integrity. Image processing and analysis were conducted using Leica LAS AF Lite software (version 1.0). Satellite cells were quantified by manual counting PAX‐7‐positive nuclei, and results were averaged across the three images. PGC‐1α quantification was assessed by calculating the mean optical density across the three fields [50, 51]. Both parameters were normalized to the number of muscle fibers within each image, with results expressed as absolute values and fold changes.

To evaluate muscle inflammatory status, approximately 50 mg of wet muscle tissue was homogenized in extraction buffer, and the concentrations of IL‐6 (DY206; assay range: 78.1–5000 pg mL^−1^), IL‐10 (DY207; assay range: 31.2–2000 pg mL^−1^), and TNF‐α (DY210; assay range: 15.6–1000 pg mL^−1^) were determined using commercial ELISA kits (DuoSet, R&D Systems, MN, USA), following the manufacturer's protocols.

### 2.3 Statistical Analyses

Statistical analyses were performed using SPSS version 23.0 (SPSS Inc., Chicago IL, USA). Data normality was assessed using the Shapiro–Wilk test. When normality was confirmed, parametric statistical procedures were applied.

A linear mixed‐effects model was used to compare performance variables (vV̇O_2_max and time to task failure [TTF]) and cardiorespiratory parameters within and between groups. For intramuscular markers (satellite cell pool and PGC‐1α optical density), the mixed linear model was applied when data were expressed as fold change. This model included time (baseline, Post‐I, and Post‐II) and group (CWI vs. control) as fixed effects, with the participants treated as a random effect. When appropriate, post hoc comparisons were conducted using the Sidak correction.

For the satellite cell pool and PGC‐1α optical density expressed as raw (unadjusted) values, analyses of covariance (ANCOVA) were conducted with group (CWI vs. control) and time (Post‐I and Post‐II) as fixed factors, using baseline values as the covariate. Sidak‐adjusted post hoc were applied when necessary.

Independent sample Student's *t*‐tests were used to compare baseline anthropometric and cardiorespiratory characteristics, as well as percentage changes from baseline (Δ%) in V̇O_2_max, vV̇O_2_max, TTF, and session load between groups. Effect sizes (Cohen's *d*) were calculated and interpreted as small (≥ 0.2), moderate (≥ 0.5), or large (≥ 0.8).

Because cytokine data were not normally distributed, nonparametric tests were applied. Friedman's test was used to assess the effect of time (i.e., baseline vs. Post‐I vs. Post‐II) on cytokine concentrations, and the Mann–Whitney *U* test was used to compare cytokine response between the control and CWI groups.

Data are presented as mean ± standard deviation (SD) for normally distributed variables and median (interquartile range) for non‐normally distributed variables. Where relevant, results are also expressed as fold change. Statistical significance was set at *p* < 0.05 for all analyses.

## 3 Results

Training adherence was 100% in both groups, with no dropouts or exclusions throughout the intervention period. Regarding training load monitoring, the accumulated internal load was 2607.7 ± 544.2 arbitrary units (a.u.) in the control group and 2558.2 ± 528.8 a.u. in the CWI group (*p* = 0.857). A significant alteration over time was observed, with training load increasing progressively over the first 4 weeks and decreasing during the final tapering week (*main effect of time*, *F* = 33.636, *p* < 0.001). A significant interaction was found for the 11th training session (*group × time effect*, *F* = 2.004, *p* = 0.021), in which the CWI group demonstrated a higher internal load compared to the control group (*post hoc p* = 0.049). No group differences were detected (*main effect of group*, *F* = 0.035, *p* = 0.853) (Figure S1).

### 3.1 Performance and Cardiorespiratory Adaptations to HIIT Program

**FIGURE 2:**
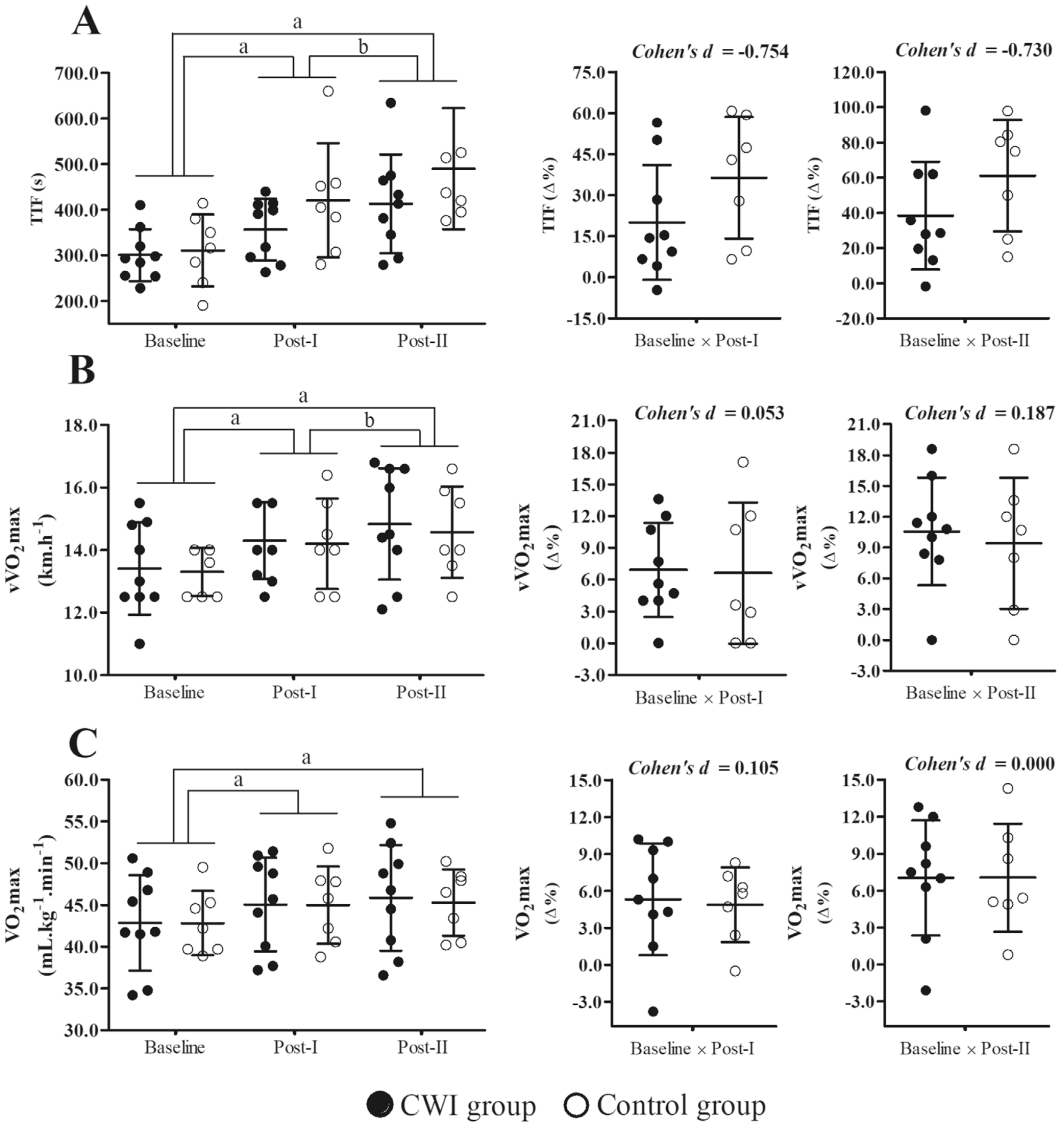
Minimal velocity at V̇O_2_max (vV̇O_2_max) (panel A), maximal oxygen uptake (V̇O_2_max) (panel B) and Time to task failure (TTF) (panel C) measured during graded exercise testing (GXT) and constant‐work‐rate (CWR) at baseline, Post‐I, and Post‐II for CWI and control groups, presented as raw data and relative to baseline values (Δ%). ^a^ *p* < 0.05 versus baseline (main effect of time). ^b^ *p* < 0.05 versus Post‐I (main effect of time).

The CWR tests were performed at 13.3 ± 0.8 km h^−1^ for the control group and 13.4 ± 1.5 km h^−1^ for the CWI group (*p* = 0.859), corresponding to 100% of the baseline vV̇O_2_max. This running speed was maintained for the subsequent evaluations at Post‐I and Post‐II. The performance outcomes of the CWR are presented in Figure 2A. A significant increase in time to task failure (TTF) was observed over time (*main effect of time*, *F* = 20.871; *p* ≤ 0.001), with improvements noted at Post‐I and Post‐II compared to baseline (*post hoc p* ≤ 0.001), as well as between Post‐I and Post‐II (*post hoc p* = 0.003). Although TTF improved across time points, no significant differences were found between groups (*main effect of group*, *F* ≤ 1.350; *p* ≥ 0.264), nor was there a significant interaction (*group × time effect*, *F* = 1.393; *p* = 0.265), whether TTF was expressed in absolute values or as Δ% (Post‐I: *p* = 0.154; Post‐II: *p* = 0.169) (Figure 2A). Although the Δ% changes from Baseline to Post‐I and Baseline to Post‐II demonstrated a moderate effect size, the corresponding comparisons did not achieve statistical significance.

Regarding the cardiorespiratory responses at exhaustion during the CWR presented in the Table 1, a significant increase in V̇O_2_ was observed (*main effect of time*, *F* = 6.402; *p* = 0.007) at Post‐I and Post‐II compared to baseline (*post hoc p* ≤ 0.048). In contrast, a significant reduction in the RER was noted (*main effect of time*, *F* = 4.323; *p* = 0.023) at Post‐I compared to baseline (*post hoc p* = 0.023). The HR also decreased significantly (*main effect of time*, *F* = 6.735; *p* = 0.004) at Post‐I compared to baseline (*post hoc p* = 0.008). Similarly, peak [La^−^] was significantly lower (*main effect of time*, *F* = 4.586; *p* = 0.021) at Post‐I compared to baseline (*post hoc p* = 0.044). However, no significant changes over time were found for other parameters at exhaustion, including absolute V̇O_2_, V̇CO_2_, V̇E, and RPE (*main effect of time*, *F* ≤ 3.490; *p* ≥ 0.053). Additionally, no significant group differences (*main effect of group, F* ≤ 0.580; *p* ≥ 0.458) or interactions (*group × time effect*, *F* ≤ 1.618; *p* ≥ 0.221) were observed for any of these variables between the control and CWI groups (Table 1).

**TABLE 1:** Exhaustion cardiorespiratory, metabolic and perceptual responses in the CWR.

	Control group			CWI group			*Interaction*		*Main effect of time*		*Main effect of group*	
	Baseline	Post‐I	Post‐II	Baseline	Post‐I	Post‐II	*F*	*p*	*F*	*p*	*F*	*p*
V̇O_2_ (mL kg^−1^ min^−1^)	42.7 ± 3.9	44.1 ± 4.5	45.1 ± 5.1	42.9 ± 5.8	44.4 ± 6.0	44.4 ± 5.4	0.378	0.690	6.402	0.007^a^ ^,^ ^b^	0.001	0.978
V̇O_2_ (mL min^−1^)	3512.8 ± 717.9	3605.5 ± 648.5	3628.0 ± 497.2	3385.3 ± 508.6	3516.5 ± 487.6	3508.4 ± 483.5	0.119	0.889	3.490	0.053	0.168	0.688
V̇CO_2_ (mL min^−1^)	4007.7 ± 762.9	3837.6 ± 741.8	3842.5 ± 715.8	3833.8 ± 626.4	3799.3 ± 550.8	3800.3 ± 557.6	18.462	0.949	2.157	0.144	0.069	0.797
V̇E (L min^−1^)	137.9 ± 16.3	135.6 ± 20.1	140.3 ± 16.7	136.8 ± 20.4	138.7 ± 24.9	139.4 ± 28.6	0.485	0.623	0.726	0.496	0.001	0.970
RER	1.14 ± 0.04	1.06 ± 0.03	1.05 ± 0.08	1.13 ± 0.05	1.08 ± 0.05	1.08 ± 0.05	0.609	0.551	4.323	0.023^a^	0.580	0.458
HR (bpm)	182.5 ± 8.8	177.8 ± 12.1	181.3 ± 7.5	183.3 ± 8.6	177.0 ± 6.7	180.1 ± 9.8	0.148	0.863	6.735	0.004^a^	0.015	0.903
Peak [La^−^] (mmol L^−1^)	9.7 ± 2.2	8.6 ± 1.7	8.6 ± 2.0	9.8 ± 1.1	9.1 ± 1.8	9.3 ± 1.4	0.491	0.618	4.586	0.021^a^	0.315	0.583
RPE (a.u.)	17.6 ± 1.3	17.1 ± 1.1	17.5 ± 1.3	17.9 ± 1.1	17.2 ± 1.4	17.8 ± 1.9	0.078	0.925	1.873	0.180	0.144	0.710

Abbreviations: CWI, cold‐water, immersion; CWR, constant work rate running effort; HR, heart rate; Peak [La^−^], peak lactate blood concentration; RER, respiratory exchange ratio; RPE, rating of perceived exertion; V̇E, ventilation; V̇O_2_, oxygen consumption.

^a^ *Main effect of time* post hoc: significant difference from baseline to Post‐I.

^b^ *Main effect of time* post hoc: significant difference from baseline to Post‐II.

Significant improvements in V̇O_2_max (*main effect of time*, *F* = 19.957, *p* < 0.001) and vV̇O_2_max (*main effect of time*, *F* = 25.474, *p* < 0.001) were observed from baseline to both Post‐I and Post‐II in GXT (*post hoc p* < 0.001). A significant difference between Post‐I and Post‐II was verified only for vV̇O_2_max (*post hoc p* = 0.032). No significant differences were found between groups (*main effect of group*, *F* ≤ 0.053, *p* ≥ 0.822), nor were there significant interactions (*group × time effect*, *F* ≤ 0.249, *p* ≥ 0.783). Additionally, no significant differences were observed in Δ% of V̇O_2_max and vV̇O_2_max between the control and CWI groups (*p* ≥ 0.697) (Figure 2B,C).

In GXT, significant increases in absolute V̇O_2_max values were observed (*main effect of time*, *F* = 15.545; *p* = 0.001) at the Post‐II moment compared to baseline (*post hoc p* = 0.001). The RER significantly decreased (*main effect of time*, *F* = 8.512; *p* = 0.002) at both Post‐I and Post‐II compared to baseline (*post hoc p* ≤ 0.034). The HR was significantly lower (*main effect of time*, *F* = 14.700; *p* = 0.001) at Post‐I compared to baseline and significantly higher at Post‐I compared to Post‐II (*post hoc p* ≤ 0.01) (Table 2). Additionally, peak lactate blood concentration decreased significantly (*main effect of time*, *F* = 631.709; *p* ≤ 0.001) at both Post‐I and Post‐II compared to baseline (*post hoc p* ≤ 0.001). Despite this result over time, no significant interactions (*group × time effect*, *F* ≤ 1.618; *p* ≥ 0.221) or groups differences (*main effects of group*, *F* ≤ 4.362; *p* ≥ 0.057) were observed between the control and CWI groups for any of these (i.e., V̇O_2_max, V̇CO_2_, V̇E, RER, HR Peak [La^−^], and RPE) variables (Table 2).

**TABLE 2:** Exhaustion cardiorespiratory, metabolic and perceptual responses in the GXT.

	Control group			CWI group			*Interaction*		*Main effect of time*		*Main effect of group*	
	Baseline	Post‐I	Post‐II	Baseline	Post‐I	Post‐II	*F*	*p*	*F*	*p*	*F*	*p*
V̇O_2_max (mL min^−1^)	3517.5 ± 648.2	3679.5 ± 632.9	3650.5 ± 557.0	3434.5 ± 524.8	3586.4 ± 456.2	3604.0 ± 435.8	0.345	0.709	15.545	0.001^b^	0.083	0.777
V̇CO_2_ (mL min^−1^)	4002.6 ± 731.7	3986.3 ± 744.7	4099.3 ± 482.3	4159.9 ± 714.7	4031.1 ± 522.4	4091.1 ± 513.8	0.543	0.591	1.559	0.240	0.046	0.834
V̇E (L min^−1^)	135.9 ± 19.8	141.6 ± 17.5	142.3 ± 9.7	140.8 ± 24.3	143.2 ± 23.6	150.5 ± 31.6	1.100	0.347	2.821	0.077	0.188	0.671
RER	1.16 ± 0.0	1.10 ± 0.0	1.14 ± 0.0	1.23 ± 0.1	1.14 ± 0.0	1.15 ± 0.0	1.392	0.268	8.512	0.002^a^ ^,^ ^b^	4.362	0.057
HR (bpm)	190 ± 9	183 ± 9	186 ± 1	191 ± 7	185 ± 5	190 ± 6	0.566	0.577	14.700	0.001^a^, ^,^ ^c^	0.385	0.545
Peak [La^−^] (mmol L^−1^)	10.0 ± 1.6	8.6 ± 1.5	9.9 ± 2.4	9.9 ± 1.6	8.9 ± 1.3	10.4 ± 1.6	0.087	0.917	631.709	0.00^a^, ^,^ ^b^	0.010	0.920
RPE (a.u)	18 ± 2	17 ± 2	18 ± 2	19 ± 1	19 ± 1	18 ± 1	1.618	0.221	0.272	0.764	0.596	0.453

Abbreviations: CWI, cold‐water; GXT, graded exercise test; HR, heart rate; Peak [La^−^], peak lactate blood concentration, immersion; RER, respiratory exchange ratio; RPE, rating of perceived exertion; V̇E, ventilation; V̇O_2_max, maximal oxygen uptake.

^a^ *Main effect of time* post hoc: significant difference from baseline to Post‐I.

^b^ *Main effect of time* post hoc: significant difference from baseline to Post‐II.

^c^ *Main effect of time* post hoc: significant difference from Post‐I to Post‐II.

### 3.2 Muscle Morphological Alterations

**FIGURE 3:**
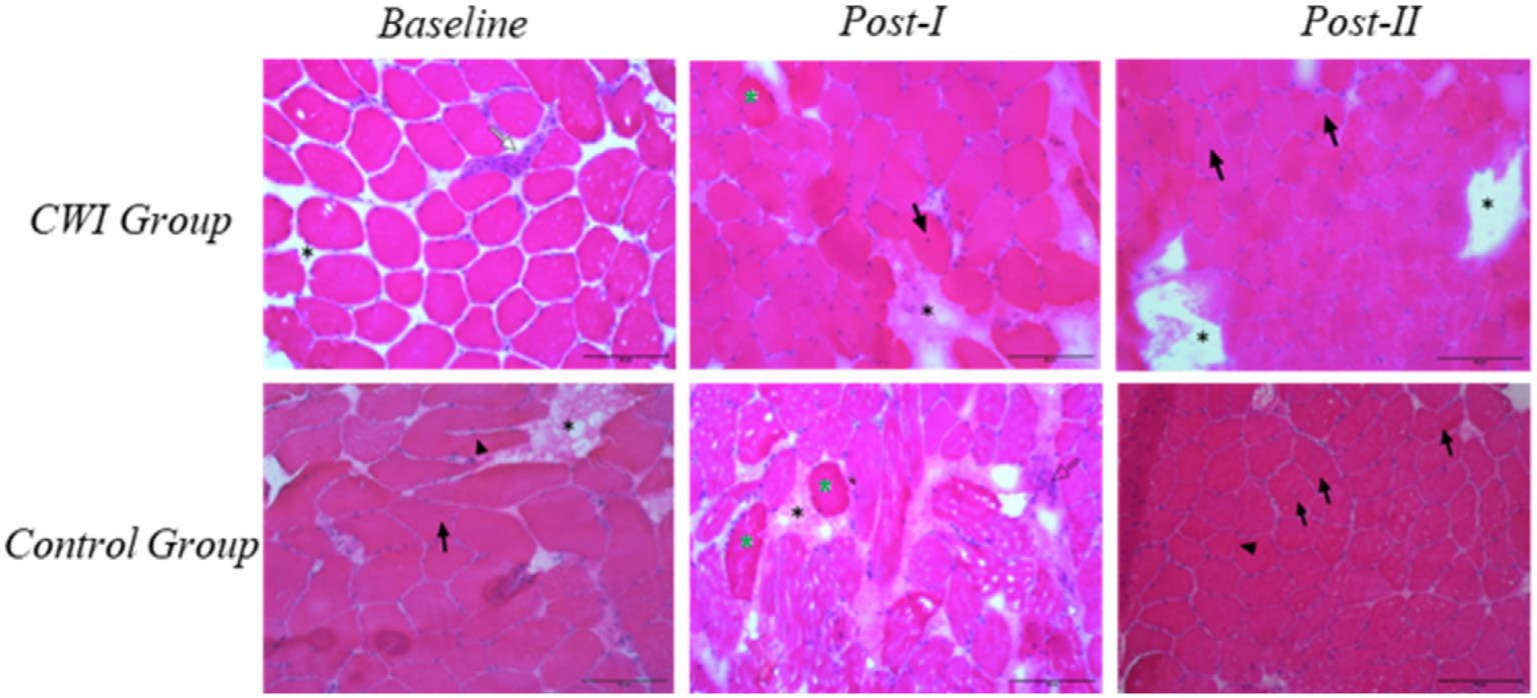
Muscle morphological alterations: representative images. CWI, cold‐water immersion; Black arrow, centralized nucleus; White arrow, inflammatory infiltrate; Black asterisk, connective tissue augmentation; Green asterisk, basophilia; Arrowhead, splitting.

It is noteworthy that the morphological alterations described below were present in approximately 5% of the muscle fibers analyzed (Table 3). In the semiquantitative analysis of muscle morphological changes, a relative temporal stability in the presence of centralized nuclei was observed in both groups. An increase in connective tissue was evident at Post‐I in both groups, with no notable differences between them. Basophilic cells' accumulation was observed only in the Control group at Post‐I. The temporal pattern of fiber splitting remained unclear. Necrosis appeared relatively stable over time in both groups, whereas a persistent inflammatory infiltrate was identified exclusively in the CWI group at Post‐II. The representative imagens of histology are presented in Figure 3.

**TABLE 3:** Muscle morphological alterations in CWI and control groups at baseline, Post‐I, and Post‐II, presented as percentages (%) and occurrence.

	Centralized nucleus % (occurrence: *yes*; *no*)	Connective tissue augmentation % (occurrence: *yes*; *no*)	Splitting % (occurrence: *yes*; *no*)	Necrosis % (occurrence: *yes*; *no*)	Basophilia % (occurrence: *yes; no*)	Inflammatory infiltrate % (occurrence: *yes; no*)
*Baseline*						
CWI group	75 (*y:6; n:2*)	38 (*y:3; n:5*)	13 (*y:1; n:7*)	13 (*y:1; n:7*)	13 (*y:1; n:7*)	25 (*y:2; n:6*)
Control group	86 (*y:6; n:1*)	29 (*y:2; n:5*)	29 (*y:2; n:5*)	0 (*y:0; n:7*)	0 (*y:0; n:7*)	29 (*y:2; n:5*)
*Post‐I*						
CWI group	63 (*y:5; n:3*)	50 (*y:4; n:4*)	38 (*y:3; n:5*)	13 (*y:1; n:7*)	13 (*y:1; n:7*)	38 (*y:3; n:5*)
Control group	100 (*y:6; n:0*)	50 (*y:3; n:3*)	17 (*y:1; n:5*)	33 (*y:2; n:4*)	50 (*y:3; n:3*)	33 (*y:2; n:4*)
*Post‐II*						
CWI group	86 (*y:6; n:1*)	43 (*y:3; n:4*)	0 (*y:0; n:7*)	0 (*y:0; n:7*)	14 (*y:1; n:6*)	43 (*y:3; n:4*)
Control group	100 (*y:7; n:0*)	14 (*y:1; n:6*)	43 (*y:3; n:4*)	14 (*y:1; n:6*)	14 (*y:1; n:6*)	14 (*y:1; n:6*)

Abbreviations: CWI, cold‐water immersion; *n*, no; *y*, yes.

### 3.3 Satellite Cell Pool and PGC‐1α Content

Using baseline raw (unstandardized) values as a covariate, a significant main effect of time was observed for satellite cell number (*F* = 9.458, *p* = 0.009; post hoc *p* = 0.003). No significant main effect of group was found (*F* = 3.180, *p* = 0.098), and no group × time interaction was observed (*F* = 1.525, *p* = 0.088).

**FIGURE 4:**
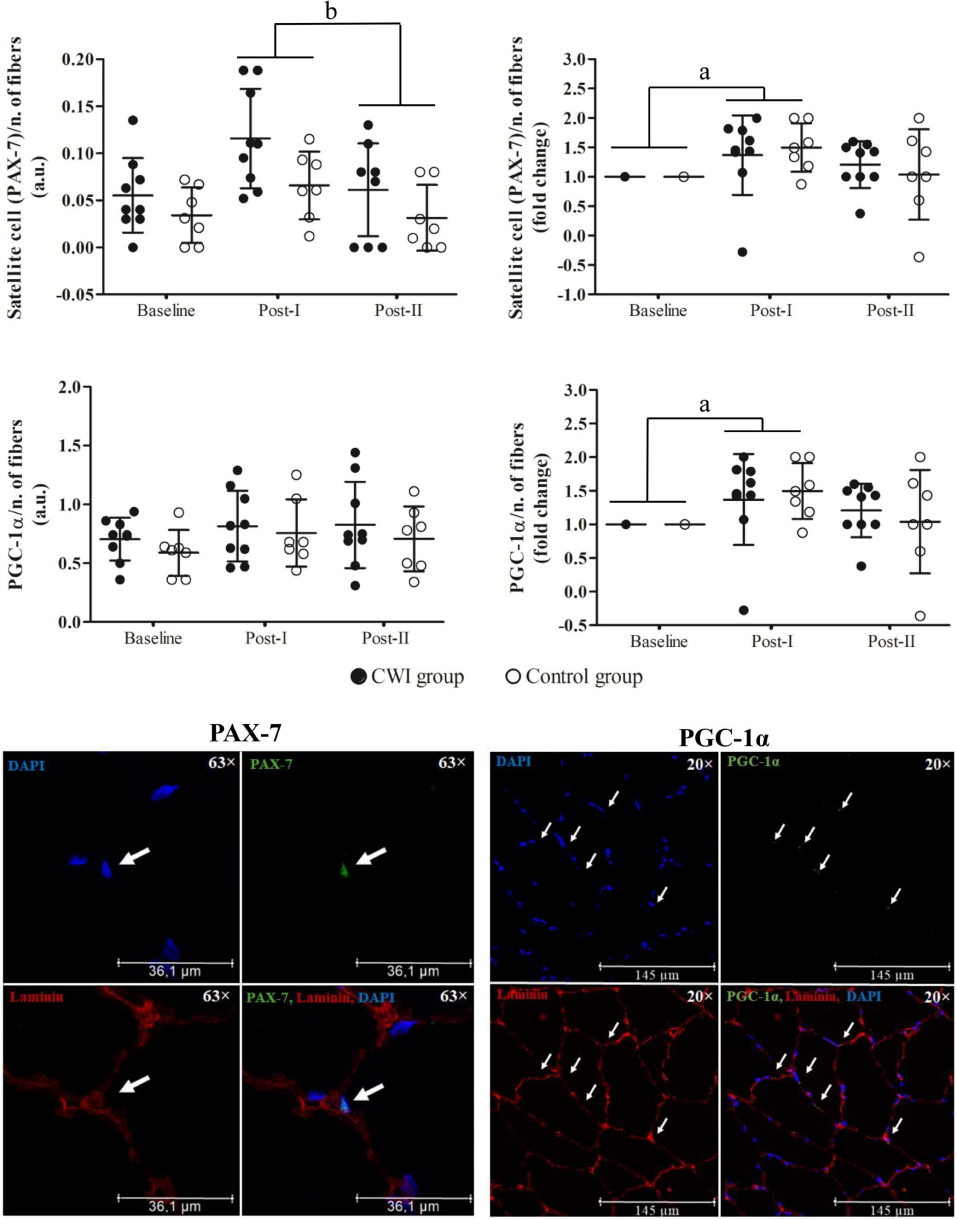
Satellite cell pool and PGC‐1α muscle content in CWI and Control groups at Baseline, Post‐I, and Post‐II, presented as values normalized by fiber number and expressed as fold change. White arrow indicates satellite cells and PGC‐1α content. CWI, cold‐water immersion; PGC‐1α, peroxisome proliferator‐activated receptor‐γ coactivator 1‐α. ^a^ *p* < 0.05 compared to baseline (*main effect of time*). ^b^ *p* < 0.05 compared to Post‐I (*main effect of time*).

When satellite cell number was expressed as fold change, a similar pattern emerged, with a significant main effect of time (*F* = 4.772; *p* = 0.018), reflecting an increase at Post‐I compared with baseline (*post hoc p* = 0.030). However, no significant effect of group (*F* = 0.009; *p* = 0.924) or group × time interaction (*F* = 0.257; *p* = 0.775) was detected (Figure 4).

For PGC‐1α content, analysis of raw values adjusted for baseline revealed no significant main effect of time (*F* = 0.022, *p* = 0.885), no effect of group (*F* = 0.053, *p* = 0.821), and no effect of group × time interaction (*F* = 0.243, *p* = 0.631).

When expressed as fold change, PGC‐1α content showed a significant main effect of time (*F* = 4.499; *p* = 0.023), with an increase at Post‐I compared with baseline (*post hoc p* = 0.036). No main effect of group (*F* = 0.282; *p* = 0.600) or group × time interaction (*F* = 0.403, *p* = 0.673) was observed (Figure 4).

### 3.4 Cytokines Muscle Contents

**FIGURE 5:**
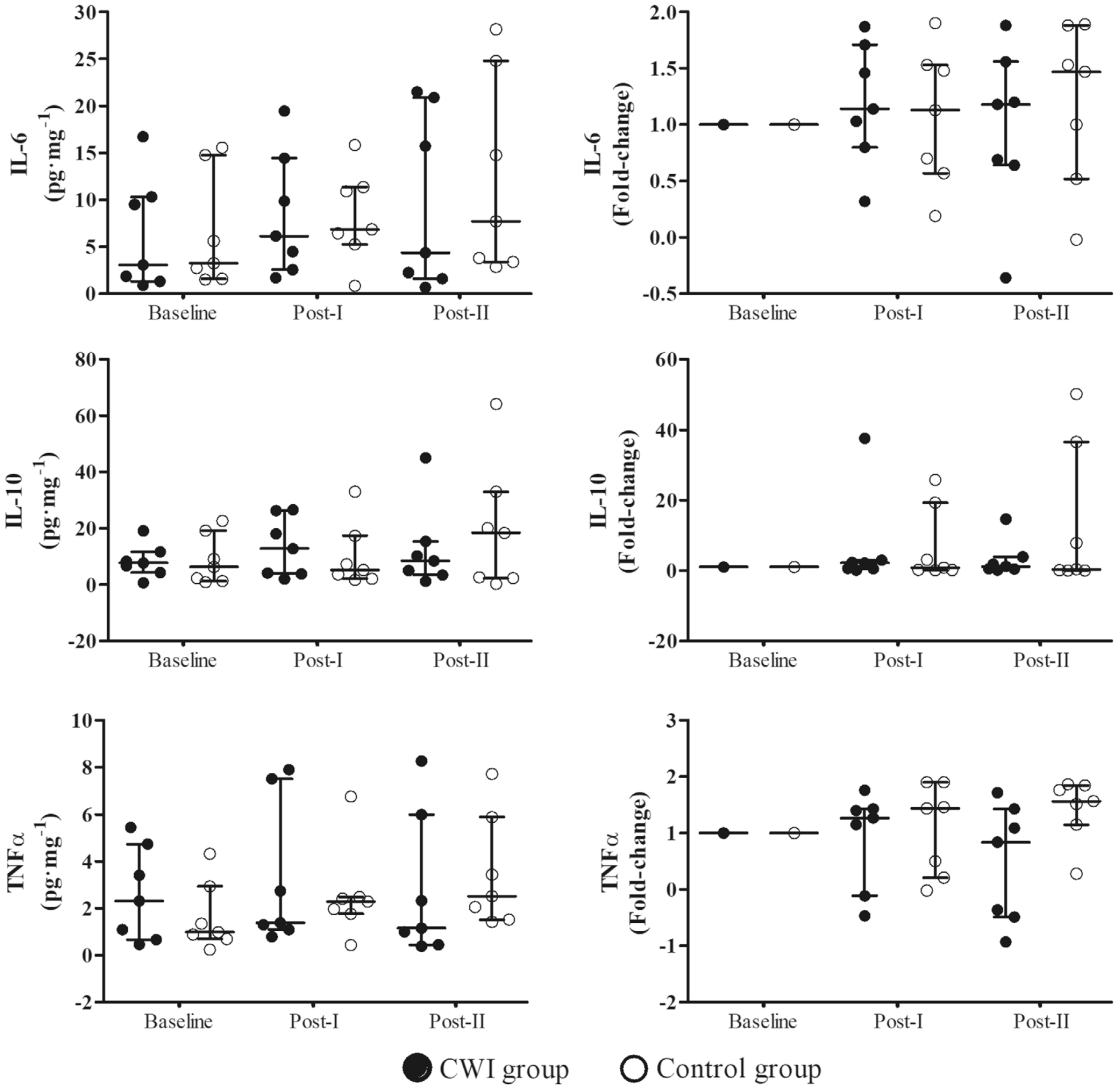
Muscle content of IL‐6, IL‐10, and TNFα in CWI and control groups at baseline, Post‐I, and Post‐II, presented as concentrations and fold changes. CWI, cold‐water immersion; IL, interleukin; TNFα, tumor necrosis factor alpha.

Over time, no significant changes were observed in muscle content of IL‐6 (*effect of time*, *x* ^2^ = 1.345; *p* = 0.510), TNFα (*effect of time*, *x* ^2^ = 1.857; *p* = 0.395), and IL‐10 (*effect of time*, *x* ^2^ = 0.462; *p* = 0.794). Similarly, no significant differences were found between groups for IL‐6 (*effect of group*, *U* = 196.000; *p* = 0.538), TNFα (*effect of group*, *U* = 213.000; *p* = 0.850), or IL‐10 (*effect of group*, *U* = 188.000; *p* = 0.566) (Figure 5).

When expressed as fold change, no significant alterations were observed over time in muscle content of IL‐6 (*effect of time*, *x* ^2^ = 1.345; *p* = 0.510), TNFα (*effect of time*, *x* ^2^ = 1.857; *p* = 0.395) or IL‐10 (*effect of time*, *x* ^2^ = 0.571; *p* = 0.751). Similarly, no group differences were found for IL‐6 (*group effect*, *U* = 219.500; *p* = 0.979), TNFα (*effect of group*, *U* = 159.000; *p* = 0.115) or IL‐10 (*effect of group*, *U* = 197.500; *p* = 0.555) (Figure 5).

## 4 Discussion

The aim of the present study was to examine the effects of 5 weeks of CWI following HIIT sessions on satellite cell pool, intramuscular cytokine content, muscle PGC‐1α expression, V̇O_2_max, and running performance. The main findings demonstrate that the regular use of CWI in this context did not influence satellite cell pool, intramuscular markers of inflammatory or mitochondrial biogenesis, cardiorespiratory fitness, or performance adaptations.

### 4.1 Performance and Cardiorespiratory Alterations

In the present study, regular use of CWI did not affect performance gains, as evidenced by lack of significant differences in both vV̇O_2_max and TTF. These findings are consistent with previous studies that investigated the effect of CWI following short‐duration high‐intensity interval training program [30], sprint interval training [24], and combined training protocols involving sprint interval training and continuous efforts on a cycle ergometer [25]. For instance, Broatch et al. [24] examined the impact of regular CWI (15 min at 10°C) following a 6‐week sprint interval training program and reported no significant effects on performance gains, including incremental test outcomes and 2‐ and 20‐km time trial performance. Similarly, Aguiar et al. [30] did not observe between‐group differences in performance improvements in the incremental test or a 15‐km time trial, although both groups demonstrated significant performance gains over time.

In this context, and as previously suggested in our review [20], the potentially detrimental effects of CWI appear to be load‐dependent, being more evident when training imposes higher mechanical stress. This effect is most apparent when CWI is applied following resistance training, which involves higher contraction intensities and greater eccentric loading, resulting in more pronounced muscle damage than HIIT. Evidence from Roberts et al. [23] indicates that CWI can suppress satellite cell activation following resistance exercise, an effect associated with attenuated strength and hypertrophy adaptations over time. Nonetheless, these interpretations should be viewed with caution, as evidence supporting load‐dependent effects of CWI remains limited.

Consistent with performance variables result, the improvement in V̇O_2_max was not affected by the regular use of CWI. In the training protocol adopted in the present study (6 to 8 efforts of 2 min at 95% of vV̇O_2_max with 2‐min passive recovery intervals), HIIT can promote adaptations at multiple physiological levels. These include enzymatic adaptations related to both aerobic and anaerobic energy metabolism at the muscular level, cardiopulmonary improvements, and, although to a lesser extent, neural adaptations [41, 42]. Indeed, despite the absence of between‐group differences, significant increases in V̇O_2_max were observed in both groups following 5 weeks of HIIT. Although this finding is not universally supported in the literature, with some studies reporting attenuated V̇O_2_max gains with CWI use [27], the majority of available evidence appears to align with our results [24, 30].

### 4.2 Morphological Changes

Muscle morphological changes were limited, affecting approximately 5% of the analyzed fibers, which warrants cautious interpretation of these findings. The relative stability of centralized nuclei and necrosis over time in both groups may suggest a low overall degree of muscle disruption [52]. The increase in connective tissue observed at Post‐I may indicate a transient remodeling response, whereas the presence of basophilic cells exclusively in the Control group suggests a detectable but limited regenerative activity [52]. Additionally, although an inflammatory infiltrate persisted at Post‐II in the CWI group, this pattern was not supported by the intramuscular cytokine data obtained in the present study [52]. Given the small proportion of affected fibers, these morphological alterations should be interpreted with caution and are unlikely to reflect the overall tissue status.

### 4.3 Satellite Cells Pool and PGC‐1α Content

Satellite cells are quiescent cells marked by PAX‐7 expression and play a key role in muscle repair and remodelling [31, 32]. It is common to observe an increase in the satellite cell pool (i.e., the number of satellite cells in the tissue) following a training program. This increase is considered an adaptive response to exercise, suggesting satellite cells activation that enhances the muscle's ability to respond to injury [33]. Importantly, the satellite cell pool can expand even in the absence of overt muscle hypertrophy, as observed in the present study. In such situations, this increase is likely related to adaptive and remodeling processes rather than to damage‐induced regeneration, since this type of training generally does not elicit substantial muscle injury [53].

Based on the findings of Roberts et al. [17], who reported acute inhibition of satellite cell activation following a session of resistance exercise combined with CWI, we initially hypothesized that repeated application of CWI would attenuate satellite cell activation over time, resulting in a smaller increase in the satellite cell pool. However, in the present study, no significant differences were found in the satellite cell pool between the control and CWI groups. This suggests that satellite cell activation persists even when CWI is performed after HIIT. Supporting this, Abreu et al. [33] proposed that endurance exercise promotes satellite cells' self‐renewal while inhibiting differentiation, likely through mechanisms involving metabolic reprogramming and respiratory inhibition. Such mechanisms may help contextualize the present findings.

Regarding markers of intramuscular adaptation, the present study showed that CWI did not affect PGC‐1α content, whereas training itself led to an increase in this protein in post‐training assessments. The elevation of PGC‐1α within the cell nucleus is known to enhance the transcription of factors involved in mitochondrial biogenesis, thereby promoting improvements in oxidative metabolism within the muscle [54]. To date, only two studies have investigated the effects of regular CWI on muscle PGC‐1α content when combined with HIIT, a training capable of influencing factors relevant to PGC‐1α synthesis (e.g., nutrient and oxygen delivery, enzymatic activity) [24, 30]. In line with our results, both studies reported no significant impact of CWI on PGC‐1α protein levels. Although some research has shown acute elevations in PGC‐1α mRNA following CWI [28], this transient molecular response does not seem to be sufficient to drive sustained increases in PGC‐1α protein content.

In contrast with these findings, some evidence in the literature suggests that CWI may reduce the acute expression of PGC‐1α mRNA, potentially mediated by systemic factors (normetanephrine concentration) [55]. For instance, one study demonstrated that immersing a single limb in cold water while keeping the contralateral limb outside resulted in decreased PGC‐1α mRNA expression in both muscles compared to a control group. This reduction was possibly linked to elevated plasmatic normetanephrine, a metabolite of norepinephrine commonly increased following CWI, which may influence local phosphorylation events within the AMPK signaling pathway, particularly at the phosphorylation of the AMPK alpha (Thr172) site of its catalytic subunit. These modifications could potentially suppress the transcriptional activity associated with PGC‐1α [32]. However, it remains unclear whether such acute inhibition has meaningful long‐term consequences. Therefore, further research is warranted to elucidate the mechanisms underlying the interaction between CWI and both the expression and content of PGC‐1α.

An important consideration when interpreting the present findings is the relative magnitude of the molecular stimuli induced by CWI compared with those elicited by high‐intensity exercise. While CWI may acutely modulate specific signaling pathways, HIIT induces a pronounced homeostatic disturbance that activates multiple, partially redundant molecular cascades related to mitochondrial biogenesis, inflammation, and muscle remodeling [56, 57, 58]. Within this context, CWI‐related molecular signals are likely insufficient to override the dominant exercise‐driven responses, which may help explain why acute molecular effects reported in some studies do not consistently translate into long‐term changes in protein content, satellite cell pool, or functional adaptations.

### 4.4 Inflammation Profile

In the present study, we investigated the effects of CWI on the intramuscular inflammatory profile by measuring the content of IL‐6, IL‐10, and TNFα. No significant changes were observed in the levels of either pro‐inflammatory or anti‐inflammatory interleukins, indicating that regular CWI use did not alter the muscle inflammatory profile. While previous studies have primarily focused on the acute effects of CWI on inflammatory responses [5, 13], to our knowledge, this is the first study to examine these effects over a longer duration. Given that satellite cell activation can be modulated by the presence of interleukins [32], we hypothesized that regular CWI would result in a cumulative reduction of these proteins, potentially leading to chronic alterations in the inflammatory environment of the muscle. However, our findings do not support this hypothesis. Although inflammatory responses play a role in the adaptations induced by HIIT [57], this type of training is known to elicit only a modest inflammatory response [59], which may partially explain the lack of significant changes observed in our study.

The primary limitation of the present study is the inability to include a placebo condition for comparison with the CWI intervention due to procedural constraints. Although placebo responses to CWI have been documented [60], the implementation of a credible sham immersion (e.g., an inert liquid presented as therapeutically active) was not feasible within the present study design. Additionally, body temperature was not monitored following CWI. Therefore, while muscle cooling was likely induced, this cannot be confirmed in the absence of direct measurements. A further limitation relates to the sample size, which may have limited statistical power for certain outcomes. Nonetheless, the experiment protocol required substantial participants commitment, including approximately 25 laboratory visits involving high‐intensity training sessions with CWI, three muscle biopsies, and multiple exhaustive tests. Consequently, although the sample size was modest, studies employing similarly demanding protocols commonly include smaller cohorts. An additional limitation concerns the study population, as only healthy, untrained men were recruited due to the specific design requirements. This choice limits generalizability of the findings, however, inclusion of other populations was not feasible. In particular, trained athletes are typically unable to suspend habitual training for an extended period, and hormonal fluctuations across the menstrual cycle could introduce additional variability in female participants.

## 5 Conclusion

In summary, 5 weeks of CWI following HIIT did not significantly affect key indicators of muscle adaptation, including the satellite cell pool, intramuscular inflammatory profile, the mitochondrial biogenesis marker (PGC‐1α), V̇O_2_max, or running performance.

## 6 Perspective

The present findings demonstrate that regular post‐exercise CWI does not attenuate intramuscular or cardiorespiratory adaptations to 5 weeks of HIIT, contradicting the initial hypothesis. These results contrast with studies reporting blunted anabolic responses during resistance training, but align with evidence showing preserved endurance performance and unchanged PGC‐1α protein levels when CWI is combined with aerobic training. By integrating intramuscular markers, inflammatory responses, and performance outcomes, the current study reinforces that CWI exerts largely neutral effects on key pathways underlying aerobic adaptations in moderately active men.

Based on these findings, routine application of CWI alongside training cannot be recommended to enhance performance adaptations, as it does not provide additional adaptive benefits. Nevertheless, these results do not challenge the well‐established use of CWI for acute symptom management, such as pain reduction, which may facilitate training tolerance in specific contexts. Future research should investigate whether these conclusions extend to other populations, longer intervention periods, or alternative CWI protocols, and should clarify how contextual factors (e.g., training intensity, training status, and recovery demands) influence the interaction between CWI and endurance adaptations.

## Author Contributions

E.S.M. and A.M.Z. conceived and designed research; E.S.M. and R.A.B.P. performed experiments; E.S.M., R.A.B.P, J.C.R.N., and E.S. analyzed data; E.S.M., R.A.B.P, J.C.R.N., W.R.B., A.S.C., and A.M.Z. interpreted results of experiments; E.S.M. prepared figures; E.S.M. and A.M.Z. drafted manuscript; E.S.M., R.A.B.P, J.C.R.N., W.R.B., A.S.C., R.A.B.P, and A.M.Z. edited and revised manuscript; E.S.M., R.A.B.P, J.C.R.N., W.R.B., A.S.C., R.A.B.P, E.S., and A.M.Z. approved final version of manuscript.

## Funding

This work was supported by The São Paulo Research Foundation (no. 2017/21724‐8), Conselho Nacional de Desenvolvimento Científico e Tecnológico (CNPq, #307556/2022‐0), and Coordenação de Aperfeiçoamento de Pessoal de Nível Superior (CAPES, #001). The authors thank Alex Castro for his assistance with the statistical analyses.

## Conflicts of Interest

The authors declare no conflicts of interest.

## Supporting information


**Figure S1:** Session internal load of each training session (1 to 12) during 5 weeks. *p < 0.05 between groups (*interaction*). CWI, cold‐water immersion. ^a–n^ *p* < 0.05 between moments (*time effect*).

## Data Availability

The data that support the findings of this study are available on request from the corresponding author. The data are not publicly available due to privacy or ethical restrictions.
