# Supplementary figures and images for: Regular Cold‐Water Immersion Following HIIT Does Not Affect Intramuscular Adaptation Markers, Inflammatory Profile or Endurance Performance
Source: Scand J Med Sci Sports. 2026 Feb 25;36(3):e70241. doi: 10.1111/sms.70241 (PMC12936277; doi:10.1111/sms.70241)

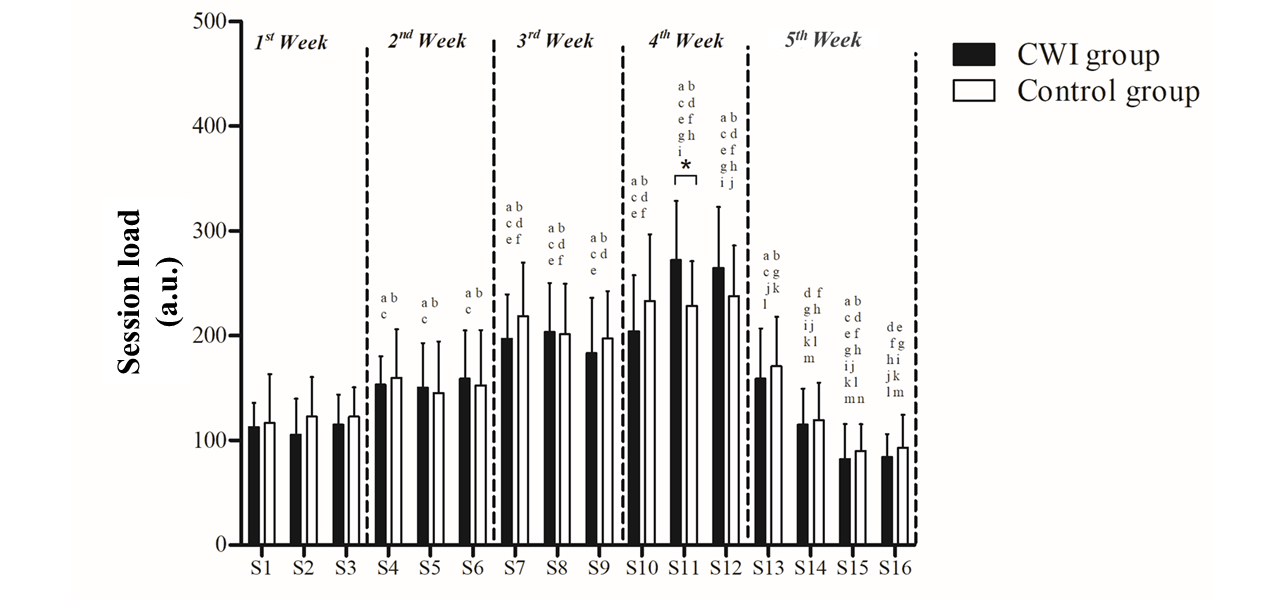

Supplement: Supplementary file 1 — Figure S1: Session internal load of each training session (1 to 12) during 5 weeks. *p < 0.05 between groups (interaction). CWI, cold‐water immersion. a–n p < 0.05 between moments (time effect). [file SMS-36-e70241-s001.tif]
